# Menopausal Transition, Body Mass Index, and Prevalence of Mammographic Dense Breasts in Middle-Aged Women

**DOI:** 10.3390/jcm9082434

**Published:** 2020-07-30

**Authors:** Eun Young Kim, Yoosoo Chang, Jiin Ahn, Ji-Sup Yun, Yong Lai Park, Chan Heun Park, Hocheol Shin, Seungho Ryu

**Affiliations:** 1 Department of Surgery, Kangbuk Samsung Hospital, Sungkyunkwan University School of Medicine, Seoul 03181, Korea; gimo.kim@samsung.com (E.Y.K.); jisup.yun@samsung.com (J.-S.Y.); yonglai.park@samsung.com (Y.L.P.); chanheun1@gmail.com (C.H.P.); 2 Center for Cohort Studies, Total Healthcare Center, Kangbuk Samsung Hospital, Sungkyunkwan University School of Medicine, Seoul 04514, Korea; jiin57.ahn@samsung.com (J.A.); hcfm.shin@samsung.com (H.S.); 3 Department of Occupational and Environmental Medicine, Kangbuk Samsung Hospital, Sungkyunkwan University School of Medicine, Samsung Main Building B2, 250, Taepyung-ro 2ga, Jung-gu, Seoul 03181, Korea; 4 Department of Clinical Research Design & Evaluation, SAIHST, Sungkyunkwan University, Seoul 06351, Korea; 5 Department of Family Medicine, Kangbuk Samsung Hospital, Sungkyunkwan University School of Medicine, Seoul 03181, Korea

**Keywords:** breast density, body-mass index, adiposity, menopause, menopausal stage, mammogram

## Abstract

The interrelationship between menopausal stage, excessive adiposity and dense breasts remains unclear. We aimed to investigate the relationship between menopausal stage and dense-breast prevalence in midlife women while considering a possible effect modification of being overweight. The present cross-sectional study comprised 82,677 Korean women, aged 35–65 years, who attended a screening exam. Menopausal stages were categorized based on the Stages of Reproductive Aging Workshop (STRAW + 10) criteria. Mammographic breast density was categorized according to Breast Imaging Reporting and Data System (BI-RADS). Dense breasts were defined as BI-RADS Breast Density category D (extremely dense). The prevalence of dense breasts decreased as menopausal stage increased (*p*-trend < 0.001), and this pattern was pronounced in overweight women than non-overweight women (*p*-interaction = 0.016). Compared with pre-menopause, the multivariable-adjusted prevalence ratios (and 95% confidence intervals) for dense breasts were 0.98 (0.96–1.00) in early transition, 0.89 (0.86–0.92) in late transition, and 0.55 (0.52–0.59) in post-menopause, among non-overweight women, while corresponding prevalence ratios were 0.92 (0.87–0.98), 0.83 (0.77–0.90) and 0.36 (0.31–0.41) among overweight women. The prevalence of dense breasts was inversely associated with increasing menopausal stages and significantly decreased from the late menopausal transition, with stronger declines among overweight women.

## 1. Introduction

Mammographic breast density refers to the amount of radiopaque epithelial and stromal tissue elements relative to the amount of radiolucent fatty elements, and is a strong predictor of breast cancer in both pre-and post-menopausal women [1,2,3]. Beast density, however, is not a static trait and is affected by various factors including age, parity, hormone use, and menopause [4,5,6]. Some studies have reported that use of combined estrogen plus progestin hormone increases breast density [7,8,9], while tamoxifen, an estrogen receptor antagonist, decreases breast density [10]. Studies also suggested that mammographic density decreases with increasing age and is strongly affected by menopause [5,6,11].

Menopause is retrospectively defined as the final menopausal period after twelve consecutive months of amenorrhea [12]. During the menopausal transition, a progressive change from pre- to post-menopause occurs, rather than simple binary states of pre- versus post-menopause; therefore, the 2011 Stages of Reproductive Aging Workshop (STRAW + 10) updated guidelines to propose a comprehensive staging system for the menopause process [12], of which transitional stages are classified as pre-menopause, early transition, late transition, and post-menopause according to menstrual bleeding patterns. During this period, hormonal changes can affect various health conditions such as vasomotor symptoms, bone loss, obesity, and cardiovascular disease (CVD) [13,14]. Few researches have addressed the association between detailed menopausal stages and mammographic breast density. Additionally, being overweight or obese is a risk factor for breast malignancy among post-menopausal women and is inversely associated with dense breasts [15,16]. Furthermore, the relationship between excessive adiposity and breast malignancy remains controversial among pre-menopausal women and some studies have even reported an inverse relationship of obesity with breast cancer risk in this age group [17,18,19]. The effects of excessive adiposity and menopausal stage on dense breasts remains unclear.

Herein, we investigated the association between menopausal stage based on the STRAW + 10 guidelines and mammographic dense-breast prevalence in midlife Korean women and we also evaluated whether this association differs by degree of adiposity, an inverse predictor of breast density.

## 2. Patients and Methods

### 2.1. Study Population

The present study is part of the Kangbuk Samsung Health Study, a cohort study of South Korean adults who participated in annual or biannual health examinations at Kangbuk Samsung Hospital Total Healthcare Centers in Seoul and Suwon, South Korea as previously described [20].

This study included 117,488 women, 35–65 years old, who received a health examination and completed questions on menopausal transition, from 2015 to 2018. A total of 26,958 participants had one or more of the following exclusion criteria: history of hysterectomy or bilateral oophorectomy (*n* = 5344); pregnancy or lactation within the past year (*n* = 5494); history of malignancy (*n* = 5857); radiation- or chemotherapy-related menopause (*n* = 653); currently taking hormone therapy (*n* = 2303); currently taking oral contraceptives (*n* = 972), mammographic finding of Breast Imaging Reporting and Data System (BI-RADS) C5 (highly suspicious of malignancy) (*n* = 5), mammographic finding of breast surgery or implants (*n* = 1224); and missing data on mammography or body-mass index (*n* = 12,959). The final sample included 82,677 participants for the analysis. 

The Institutional Review Board of Kangbuk Samsung Hospital approved this study (IRB No. KBSMC 2019-05-035) and waived the requirement for informed consent because of the access to non-identified retrospective data routinely obtained during the health check-up program.

### 2.2. Data Collection

Data pertaining to demographic characteristics, health behaviors, physical activity, dietary intake, medical history, and reproductive factors (parity, menstrual patterns, age at menarche, and use of hormones or oral contraceptives) were identified via a standardized, structured, self-administered questionnaire [20,21]. Parity was defined as the number of pregnancies including live births and stillbirths. According to the STRAW + 10 criteria, menopausal stages were classified as (1) pre-menopause; (2) early menopausal transition (a persistent difference of ≥7 days in menstrual cycle length changes); (3) late menopausal transition (an interval of amenorrhea ≥60 days); and (4) post-menopause (amenorrhea for ≥12 months) [12]. Physical activity level was categorized as inactive, minimally active, or health-enhancing physical activity (HEPA) [22]. HEPA was defined as physical activity that meets either of two criteria: (1) vigorously intense activity on three or more days per week accumulating ≥1500 metabolic equivalent (MET)-minutes/week (1 MET is energy expenditure at rest); or (2) seven days of any combination of walking, moderate intensity, or vigorous intensity activities achieving at least 3000 MET min/week [22].

Measurements of sitting blood pressure (BP) and anthropometric factors were performed by trained nurses. Weight was measured to the nearest 0.1 kg on a bioimpedance analyzer (Inbody 720; Biospace Co., Seoul, Korea) which was validated for reproducibility and accuracy of body composition measurements [23]. Body mass index (BMI) was categorized based criteria for Asian populations: [24] underweight, below 18.5 kg/m^2^; normal weight, 18.5 to 23 kg/m^2^; overweight, 23 to 25 kg/m^2^; and obese, ≥25 kg/m^2^. Abdominal obesity was defined as waist circumference ≥85 cm as criteria specific for Korean women [25,26]. Hypertension was defined as BP ≥ 140/90 mmHg, or currently taking anti-hypertensive medication.

Fasting blood sample was used to measure glucose, insulin, high-sensitivity C-reactive protein (hsCRP) and lipid profiles. Insulin resistance was estimated using Homeostatic Model Assessment of Insulin Resistance (HOMA-IR) (fasting blood insulin (mU/mL) × fasting blood glucose (mmol/L)/22.5)). Diabetes was defined as fasting serum glucose ≥ 126 mg/dL, hemoglobin A1c ≥ 6.5%, or currently taking glucose-lowering agents or insulin.

Mammograms (standard craniocaudal and mediolateral oblique views of bilateral breasts) were obtained with a full-field digital mammography system (Selenia Dimension, HOLOGIC, Marlborough, MA, USA; Senographe DS, General Electric Company, Milwaukee, WI, USA). All mammography was interpreted and reported by a total of nine experienced breast-imaging radiologists who were blinded to the aims of this study. According to the American College of Radiology BI-RADS, breast density was categorized as almost entirely fat (≤25% fibroglandular tissue), scattered fibroglandular (26–50%), heterogeneously dense (51–75%), or extremely dense (>75%) [27]. For this study, dense breasts were defined as breast density that is “extremely dense,” which is a definition that has been used in other studies [28].

Fatty liver based on abdominal ultrasound (US) was diagnosed by experienced radiologists using standard criteria such as the presence of a diffuse increase in fine echoes in the liver parenchyma compared with kidney or spleen parenchyma, deep beam attenuation, and bright vessel walls [29].

The data are not available to be shared publicly outside of the Kangbuk Samsung Hospital as we do not have IRB permission for distributing the data. Analytical methods are available from the corresponding author on reasonable request.

### 2.3. Statistical Analyses

Participant characteristics are presented as descriptive summary statistics according to menopausal stage. For linear trends, menopausal stage was treated as a continuous variable in regression models.

The association between dense-breast prevalence and menopausal stage was assessed with a Poisson regression model, and prevalence ratios (PR) and 95% confidence internals (CI) were estimated for dense breasts across menopausal stages while referring to pre-menopause as the reference category. The first model was adjusted for age and then further adjusted for study center, year of exam, education attainment (≤12 years, >12 years, or unknown), smoking status (never, past, current, or unknown), alcohol intake (0, <10, ≥10 g/day, or unknown), physical activity (inactive, minimally active, HEPA, or unknown), BMI (continuous), total energy intake (in quintiles or missing), age at menarche (continuous), and parity (none, 1 to 2, ≥3 or unknown).

We also performed subgroup analyses that were stratified by waist circumference (<85 cm vs. ≥85 cm), body-fat percentage (<30% vs. ≥30%), fatty liver (no vs. yes), smoking (never smokers vs. ever smokers), daily average alcohol consumption (<10 g vs. ≥10 g), health enhancing physical activity (no vs. yes), insulin resistance defined as HOMA-IR ≥2.5 (no vs. yes), and hsCRP (<1.0 mg/L vs. ≥1.0 mg/L). Interactions by subgroup were assessed using likelihood ratio tests comparing models with and without multiplicative interaction terms.

STATA, version 16.0 (StataCorp LP, College Station, TX, USA) was used for statistical analyses. The two-tailed *p*-value < 0.05 was considered statistically significant.

## 3. Results

Characteristics of 82,677 women were compared according to their breast density (Table 1). The prevalence of women in pre-menopause, early transition, late transition, and post-menopause was 56.3%, 16.8%, 7.6%, and 19.3%, respectively. The prevalence of extremely dense breasts was 39.4% overall, 47.5% in pre-menopause, 45.0% in early transition, 34.4% in late transition and 13.1% in post-menopause. Women with dense breasts tend to be younger, taller, more educated, less physically active, less post-menopausal, have lower prevalence of fatty liver, hypertension, and diabetes, and have lower levels of BMI, BP, glucose, HOMA-IR, hsCRP, total cholesterol, low-density lipoprotein cholesterol (LDL-C), and triglycerides.

**Figure 1:**
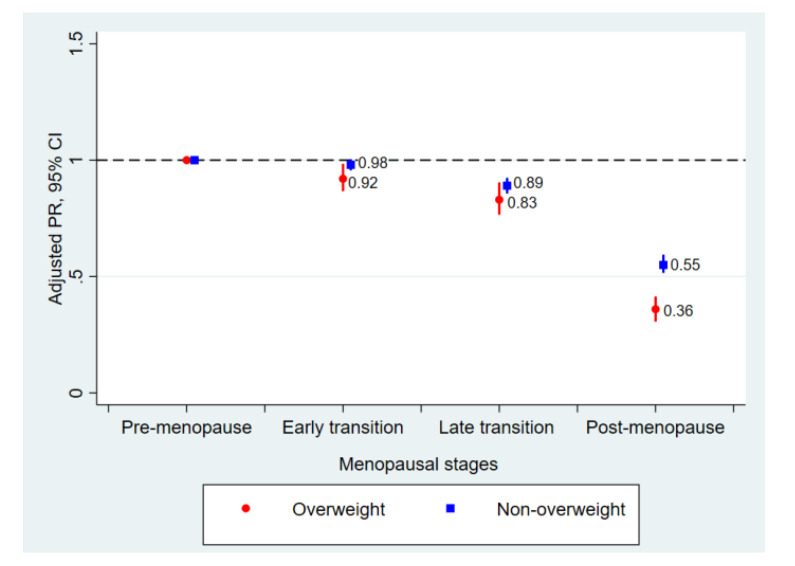
Prevalence ratios of dense breast across menopausal stage by the presence of overweight.

Table 2 presents dense-breast prevalence across all menopausal stages. The prevalence of dense breasts decreased with increasing menopause stage in both overweight and non-overweight participants (*P*-trend < 0.001), but the inverse relationship was more pronounced among overweight women than non-overweight women (*P*-interaction by being overweight = 0.016) (Table 2 and Figure 1). After adjusting for age and possible confounders, the multivariable-adjusted PRs (95% CIs) for dense-breast prevalence comparing early transition, late transition, and post-menopause to pre-menopause were 0.98 (0.96–1.00), 0.89 (0.86–0.92), and 0.55 (0.52–0.59), respectively, in non-overweight women, and 0.92 (0.87–0.98), 0.83 (0.77–0.90), and 0.36 (0.31–0.41) in overweight women. When we adjusted for body fat percent instead of BMI, the results were similar.

In subgroup analyses, the inverse relationship between menopausal stage and dense breasts was pronounced among women with higher waist circumference (≥85 cm vs. <85 cm), higher body-fat percentage (≥30% vs. <30%), fatty liver (yes vs. no) and insulin resistance (Table 3). Multivariable adjusted PRs (95% CIs) for dense breasts comparing early transition, late transition, and post-menopause to pre-menopause were 0.97 (0.95–0.99), 0.94 (0.90–0.98), and 0.63 (0.59–0.67), respectively, in women with body-fat percentage <30%, and 0.97 (0.93–1.00), 0.83 (0.79–0.88), and 0.43 (0.40–0.48) in women with body-fat percentage ≥30% (*p* for interaction <0.001). With respect to smoking status; multivariable-adjusted PRs (95% CI) for dense breasts comparing early transition, late transition, and post-menopause to pre-menopause were 0.96 (0.94–0.97), 0.89 (0.86–0.92), and 0.48 (0.45–0.50), respectively, in never smokers (*p*-trend < 0.001), and 1.07 (0.94–1.22), 1.09 (0.88–1.36), and 0.74 (0.54–1.01) in current smokers (*p*-trend = 0.158) (*p*-interaction by smoking = 0.258) (Table 3 and Table A1 Appendix A). The relationship of menopausal stage with dense breasts did not significantly differ by alcohol consumption, physical activity, or low-grade inflammation defined hsCRP ≥ 1.0 mg/L.

## 4. Discussion

In the present large-scale sample of 82,677 midline women, menopausal stage was inversely related to the prevalence of dense breasts in a dose-response relationship and significant decline in prevalence was noted from the late menopausal transition compared with pre-menopause. The absolute prevalence of dense breasts was lower in overweight women (BMI ≥ 23 kg/m^2^) across all menopausal stages, but the relative reduction in dense-breast prevalence across menopausal stages was greater in overweight women than non-overweight women. A similar pattern was also observed when body-fat percentage ≥30%, abdominal obesity based on waist circumference or fatty liver were used as an indicator of excessive adiposity instead of BMI measures. Our study findings indicate that dense-breast prevalence significantly decreases from pre-menopause to late menopause and degree of decline is greater with presence of excessive adiposity.

Consistent with our result, prior studies have reported an inverse association between menopause and breast density [4,30]. In accordance with ours, the prevalence of mammographic dense-breast tissue has been reported to decline with increasing age, with a marked decrease occurring during menopause [6]. In line with our results, Boyd et al. [4] also reported a greater decline in breast density among women who experienced a relatively early natural menopause compared with age-matched controls who maintained a pre-menopausal status during the same follow-up duration. A recent collaborative-pooled analysis of cross-sectional studies from 22 countries also agreed with our findings estimating that post-menopausal women had 3.5 cm^2^ less mean dense area than pre-menopausal women [31]. However, all these studies included menopausal status as a binary category (pre- vs. post-menopausal status) without differentiating the menopausal transition stages. In our study, which uses the updated menopausal stages (STRAW + 10) [12], dense-breast prevalence decreased with increasing menopausal stage and a significant acceleration of the decrease was observed at the late transition stage. Indeed, the menopausal transition is the period before the final menstrual period and is characterized by hormonal changes such as an increase in follicle stimulating hormone and a decline in estradiol [32]. A population-based cohort study reported that serum estradiol levels are relatively stable between −10 and −2 years to the final menstrual period, while a rapid decrease in estradiol begins about 2 years prior to the final menstrual period [33]; thus, estradiol changes prior to menopause may support our findings.

Notably, in our study, the decreasing pattern of dense-breast prevalence across menopausal stages was blunted in current smokers. The interrelationship between smoking, dense breast, and breast cancer remains controversial, but several studies reported an inverse relationship between active smoking and breast density, supporting the hypothesis that smoking may exert an anti-estrogenic influence on breast tissue [34,35,36]. Blood levels of estradiol and estrone during oral hormonal treatment in smokers have been shown to reach only half the concentration of those in non-smokers, and cigarette smoking appears to play an anti-estrogenic role on breast tissue [35]; thus, the anti-estrogenic effect of smoking might partly explain the attenuated association between menopausal stage and dense breasts since estradiol concentrations might have been lower in smokers prior to menopausal transition.

As aspects of the influence of BMI on breast density, the association between being overweight or obese and dense breast was addressed in multiple studies. Maskarinec et al. [37] reported that being overweight was related to lower breast density at baseline; however, the declines in breast density over time were significantly slower among overweight women (BMIs > 25 kg/m^2^). Engmann et al. stated that high pre-menopausal dense-tissue volume was a strong predictor of greater reductions in volume across the menopausal transition [38]. They found no differences in reduction of dense-tissue volume over time by BMI [38]. Another recent study of 24,556 women by Hart et al. showed that BMI calculated using self-report of height and weight was inversely associated with percent dense-tissue volume and an increasing BMI had a longitudinal relationship with volume decrease and percent dense-tissue volume among pre- and post-menopausal women [39].

In our study, the absolute prevalence of dense breasts was much lower in overweight women than non-overweight women and this pattern was observed at each menopausal stage. However, the relative reduction in prevalence from pre- to post-menopause was more evident in overweight women than non-overweight women. The reason for these observations is not yet clear, but several explanations are possible. Increased BMI was longitudinally associated with a decline in dense-tissue volume and percent volume regardless of menopausal status [40]. Additionally, studies have demonstrated that BMI was strongly positively correlated with non-dense areas or non-dense volume and moderately inversely associated with dense areas or dense volume [41,42,43]. Thus, in our study, a greater decline in dense breasts, reflective of the relative percentage of fibroglandular tissue compared with fat tissue, especially in overweight women, might be explained by a combination of the inverse relationship of BMI with dense tissue and the positive effect of BMI on non-dense tissue. However, our result showing greater decline in density across menopausal stages, especially in overweight patients, is contradictory because obesity in post-menopausal women and dense breasts are known risk factors for breast malignancy. Indeed, the role of breast adipose tissue in breast malignancy risk is not currently clear but fat tissue might provide a microenvironment that promotes carcinogenesis through different mechanisms, including chronic inflammation [44,45]. Growth-hormone-induced increase in free fatty acids release from adipocytes and an increase in the lipid substrate and resultant oxidative injury also have been proposed [44,46]. Because of the use of multiple comparisons, the observed difference might occur by chance between never smokers and ever smokers or between overweight and non-overweight women. Future researches are required to understand the underlying mechanisms of the possible modifying effect of smoking and excessive adiposity on the relationship of menopausal stage with breast density.

Several limitations should be noted in the present study. First, the cross-sectional design precludes identifying a temporal and causal association between menopausal stage, BMI, and dense breasts. Second, the BI-RADS scores measured density qualitatively, not with digital quantification. However, qualitative density assessment by radiologists has been reported to yield reasonable and moderate agreement with automated software measurements and is widely used in clinical settings [47]. Third, detailed information on type of female hormone therapy and use duration were not available for this study, limiting our ability to evaluate the influence of female hormone use on the relationship of menopausal stage with breast density. Fourth, intra- and inter-observer reliability tests among breast imaging radiologists were not performed in this study. Previous studies from other groups have reported acceptable agreement between BI-RADS scores by different radiologists with 80.9% agreement and a kappa of 0.77 (0.76–0.79) [1,2]. In our study, different breast imaging radiologists were involved in mammographic interpretation and reporting over time; however, they were unaware of the study aims. We included the year of the questionnaire and study center as a covariate in the multivariable models, which did not alter the results. However, some degree of residual confounding related to measurement errors and other unmeasured confounding factors cannot be excluded in observed associations between menopausal stage and breast density. Finally, the present study population comprised apparently healthy, middle-aged, highly educated Korean women who regularly received health-screening examinations; thus, findings might not be generalizable to other populations with different age and race/ethnicity characteristics. However, our study also has many strengths, including its large sample size, use of detailed menopausal stages, standardized clinical measurements and imaging, and inclusion of a wide range of confounders, enabling us to demonstrate an independent relationship of menopausal stage with breast density with a possible modifying effect of BMI on these associations.

## 5. Conclusions

In the present, large-scale sample of midlife Korean women, the prevalence of dense breasts significantly and independently decreased from the late transition of menopausal stage. This association was consistently observed in both overweight and non-overweight women but was more pronounced in overweight women. Further research using a longitudinal design and sophisticated measurements for breast-tissue density are required to determine the interrelationships of BMI, menopausal stage, and breast density, and such findings may help better understand the pathophysiology of breast cancer.

## Figures and Tables

**Table 1:** Participant characteristics according to dense versus non-dense breasts (*n* = 82,677).

Characteristic	Dense Breasts		*p*-Value
	Absent	Present	
Number	50,080	32,597	
Age (years) ^a^	45.8 (8.0)	41.6 (5.0)	<0.001
Height (m) ^a^	159.6 (5.3)	160.7 (5.0)	<0.001
BMI (kg/m^2^) ^a^	23.2 (3.2)	20.9 (2.4)	<0.001
BMI category (kg/m^2^)			<0.001
<18.5 (underweight)	3.0	13.0	
18.5–22.9 (normal weight)	51.0	70.2	
23–24.9 (overweight)	21.9	10.8	
≥25 (obesity)	24.1	6.1	
Body-fat percentage (*n* = 82,633)	32.5 (5.7)	28.0 (5.4)	<0.001
Waist circumference (cm) (*n* = 82,659)	78.7 (8.3)	72.5 (6.5)	<0.001
Fatty liver on ultrasound (%) (*n* = 82,267)	23.8	5.9	<0.001
Smoking status (*n* = 80,826)			0.369
Never smokers, (%)	95.6	95.8	
Former smokers, (%)	2.5	2.3	
Current smokers, (%)	1.9	1.9	
Alcohol intake ≥10 g/day, (%) ^c^ (*n* = 77,407)	12.3	12.2	0.796
HEPA, (%) (*n* = 82,441)	14.9	12.1	<0.001
Higher education, (%) ^d^ (*n* = 80,664)	70.8	82.2	<0.001
Hypertension, (%) (*n* = 82,671)	10.3	3.0	<0.001
Diabetes mellitus, (%) (*n* = 82,670)	4.6	1.1	<0.001
Medication for hyperlipidemia, (%)	5.1	1.0	<0.001
Family history of breast cancer (%)	3.3	3.3	0.661
Early menarche (%) ^e^ (*n* = 82,274)	4.5	4.3	0.049
Parity number (%) (*n* = 79,130)			<0.001
0	7.8	13.8	
1–2	78.1	79.7	
≥3	14.1	6.6	
Female hormone medication (%)	1.6	1.7	0.382
Systolic BP (mmHg) ^a^ (*n* = 82,671)	107.2 (12.8)	102.1 (10.3)	<0.001
Diastolic BP (mmHg) ^a^ (*n* = 82,671)	68.1 (9.3)	65.3 (8.1)	<0.001
Glucose (mg/dL) ^a^	95.0 (15.1)	91.4 (9.8)	<0.001
Total cholesterol (mg/dL) ^a^	194.7 (34.7)	186.2 (30.9)	<0.001
LDL-C (mg/dL) ^a^ (*n* = 82,504)	125.4 (33.4)	113.9 (28.6)	<0.001
HDL-C (mg/dL) ^a^	64.8 (16.0)	70.0 (15.6)	<0.001
Triglycerides (mg/dL) ^b^	81 (60–113)	69 (54–91)	<0.001
hsCRP (mg/L) ^b^ (*n* = 57,460)	0.4 (0.3–0.8)	0.3 (0.2–0.5)	<0.001
HOMA-IR ^b^ (*n* = 81,649)	1.32 (0.88–1.98)	1.11 (0.76–1.58)	<0.001
Total calorie intake (kcal/day) ^b^ (*n* = 47,947)	1131 (812–1496)	1092 (789–1444)	<0.001

Data are expressed as ^a^ mean (standard deviation), ^b^ median (25th percentile–75th percentile), or percentage; if participants had missing values for a variable, the number of participants without missing data for that variable are presented in the parenthesis; otherwise, the number of participants without missing data for each value indicates all participants (*n* = 82,677); ^c^ ≥10 g of ethanol per day, ^d^ ≥college graduate, ^e^ age at first menstrual period <12 years; abbreviations: BMI; body-mass index, BP; blood pressure, CVD; cardiovascular disease, HDL-C; high-density lipoprotein cholesterol, hsCRP; high-sensitivity C-reactive protein, HEPA; health-enhancing physical activity, HOMA-IR; homeostasis model assessment of insulin resistance, LDL-C; low-density lipoprotein cholesterol.

**Table 2:** Dense-breast prevalence according to menopausal stage among overall, overweight, and non-overweight women.

	Menopausal Stages				*p* for Trend
	Pre-Menopause	Early Transition	Late Transition	Post-Menopause	
Overall population					
No.	46,532	13,896	6287	15,962	
Cases of dense breasts (%)	47.5	45.0	34.4	13.1	
Age-adjusted PR (95% CI)	reference	0.97 (0.95–0.99)	0.84 (0.81–0.87)	0.48 (0.46–0.51)	<0.001
Multivariable-adjusted PR (95% CI) ^a^	reference	0.96 (0.94–0.98)	0.89 (0.86–0.92)	0.48 (0.46–0.51)	<0.001
Non-overweight (BMI < 23 kg/m^2^)					
No.	32,772	9660	3690	8005	
Cases of dense breasts (%)	56.6	54.7	45.0	20.1	
Age-adjusted PR (95% CI)	reference	0.98 (0.96–1.00)	0.89 (0.86–0.92)	0.54 (0.51–0.57)	<0.001
Multivariable-adjusted PR (95% CI) ^a^	reference	0.98 (0.96–1.00)	0.89 (0.86–0.92)	0.55 (0.52–0.59)	<0.001
Overweight (BMI ≥ 23 kg/m^2^)					
No.	13,760	4236	2597	7957	
Cases of dense breasts (%)	25.8	23.0	19.3	6.1	
Age-adjusted PR (95% CI)	reference	0.90 (0.85–0.96)	0.83 (0.76–0.90)	0.35 (0.30–0.40)	<0.001
Multivariable-adjusted PR (95% CI) ^a^	reference	0.92 (0.87–0.98)	0.83 (0.77–0.90)	0.36 (0.31–0.41)	<0.001

A Poisson regression model with robust variance was used. *p* for interaction by being overweight = 0.016 in the multivariable-adjusted model; ^a^ adjusted for age, center, year of examination, educational level, smoking status, alcohol consumption, physical activity level, total energy intake, body-mass index (for overall population), parity, and age at menarche.

**Table 3:** Dense-breast prevalence ratios according to menopausal stage in clinically relevant subgroups.

Subgroup	Menopausal Stages				*p* for Trend	*p* for Interaction
	Pre-Menopause	Early Transition	Late Transition	Post-Menopause		
Waist circumference						0.001
<85 cm (*n* = 70,752)	reference	0.98 (0.96–1.00)	0.88 (0.85–0.91)	0.53 (0.51–0.56)	<0.001	
≥85 cm (*n* = 11,907)	reference	0.84 (0.74–0.95)	0.84 (0.72–0.97)	0.31 (0.24–0.41)	<0.001	
Body-fat percentage						<0.001
<30% (*n* = 37,730)	reference	0.97 (0.95–0.99)	0.94 (0.90–0.98)	0.63 (0.59–0.67)	<0.001	
≥30% (*n* = 44,903)	reference	0.97 (0.93–1.00)	0.83 (0.79–0.88)	0.43 (0.40–0.48)	<0.001	
Fatty liver on ultrasound						<0.001
No (*n* = 68,481)	reference	0.97 (0.95–0.98)	0.90 (0.87–0.93)	0.52 (0.49–0.54)	<0.001	
Yes (*n* = 13,786)	reference	0. 87 (0.78–0.96)	0.85 (0.75–0.97)	0.38 (0.31–0.46)	<0.001	
Smoking status						0.258
Never smokers (*n* = 79,359)	reference	0.96 (0.94–0.97)	0.89 (0.86–0.92)	0.48 (0.45–0.50)	<0.001	
Former smokers (*n* = 1936)	reference	0.93 (0.83–1.05)	0.83 (0.65–1.06)	0.59 (0.42–0.85)	0.002	
Current smokers (*n* = 1531)	reference	1.07 (0.94–1.22)	1.09 (0.88–1.36)	0.74 (0.54–1.01)	0.158	
Alcohol intake						0.347
<10 g /day (*n* = 67,913)	reference	0.95 (0.93–0.97)	0.89 (0.86–0.93)	0.49 (0.46–0.52)	<0.001	
≥10 g/day (*n* = 9494)	reference	0.98 (0.93–1.03)	0.85 (0.77–0.94)	0.50 (0.43–0.58)	<0.001	
HEPA						0.200
No (*n* = 71,072)	reference	0.95 (0.93–0.97)	0.88 (0.85–0.92)	0.48 (0.45–0.51)	<0.001	
Yes (*n* = 11,369)	reference	1.01 (0.96–1.07)	0.95 (0.86–1.05)	0.52 (0.46–0.59)	<0.001	
HOMA-IR						<0.001
<2.5 (*n* = 72,436)	reference	0.96 (0.94–0.98)	0.89 (0.86–0.92)	0.50 (0.48–0.53)	<0.001	
≥2.5 (*n* = 9213)	reference	0.91 (0.83–0.99)	0.86 (0.75–0.98)	0.34 (0.27–0.43)	<0.001	
HsCRP						0.354
<1.0 mg/L (*n* = 47,548)	reference	0.96 (0.94–0.98)	0.92 (0.88–0.95)	0.50 (0.47–0.54)	<0.001	
≥1.0 mg/L (*n* = 9912)	reference	1.01 (0.94–1.08)	0.89 (0.80–1.00)	0.48 (0.39–0.59)	<0.001	

A Poisson regression model with robust variance was used. The multivariable model was adjusted for age, body-mass index (except for waist circumference and body fat percentage), center, year of examination, educational level, smoking status, alcohol consumption, physical activity, total energy intake, parity, and age at menarche.

## Abbreviations

BMI	Body mass index
BI-RADS	Breast Imaging Reporting and Data System
BP	Blood pressure
CIs	Confidence intervals
CVD	Cardiovascular disease
HEPA	Health-enhancing physical activity
HOMA-IR	Homeostasis model assessment of insulin resistance
hsCRP	High sensitive C-reactive protein
LDL-C	Low-density lipoprotein cholesterol
PR	Prevalence ratio

## Appendix A

**Table A1:** Dense-breast prevalence according to menopausal stage among never, former and current smokers (*n* = 80,826).

	Menopausal Stages				*p* for Trend
	Pre-Menopause	Early Transition	Late Transition	Post-Menopause	
Never-smokers					
No.	44,443	13,177	5997	13,742	
Cases of dense breasts (%)	47.7	45.1	34.4	13.3	
Age-adjusted PR (95% CI)	reference	0.96 (0.94–0.99)	0.84 (0.81–0.87)	0.48 (0.45–0.51)	<0.001
Multivariable-adjusted PR (95% CI) ^a^	reference	0.96 (0.94–0.97)	0.89 (0.86–0.92)	0.48 (0.45–0.50)	<0.001
Former smokers					
No.	1201	402	146	187	
Cases of dense breasts (%)	42.3	41.8	28.8	17.1	
Age-adjusted PR (95% CI)	reference	0.99 (0.87–1.13)	0.73 (0.56–0.95)	0.62 (0.41–0.93)	0.005
Multivariable-adjusted PR (95% CI) ^a^	reference	0.93 (0.83–1.05)	0.83 (0.65–1.06)	0.59 (0.42–0.85)	0.002
Current smokers					
No.	838	303	120	270	
Cases of dense breasts (%)	43.4	47.5	40.0	15.9	
Age-adjusted PR (95% CI)	reference	1.11 (0.96–1.28)	1.03 (0.82–1.30)	0.68 (0.48–0.96)	0.25
Multivariable-adjusted PR (95% CI) ^a^	reference	1.07 (0.94–1.22)	1.09 (0.88–1.36)	0.74 (0.54–1.01)	0.158

A Poisson regression model with robust variance was used. *p* for interaction by smoking status = 0.258 in the multivariable-adjusted model; ^a^ adjusted for age, center, year of exam, education attainment, alcohol intake, physical activity, total energy intake, body-mass index, parity, and age at menarche.
